# Validation of rectal swabbing for total and aerobic gut microbiota study

**DOI:** 10.1128/spectrum.01823-24

**Published:** 2025-02-19

**Authors:** Julie Marin, Paul Albin Bertoye, Andre Birgy, Samira Dziri, Mathilde Lescat

**Affiliations:** 1 Université Sorbonne Paris Nord and Université Paris Cité, Inserm, IAME, Bobigny, France; 2 Service Microbiologie, AP-HP, Hôpital Avicenne, Bobigny, France; 3 Service Microbiologie, AP-HP, Hôpital Robert-Debré, Paris, France; 4 Université Paris Cité, Inserm, Institut Cochin, Paris, France; Quest Diagnostics Nichols Institute, Chantilly, Virginia, USA

**Keywords:** digestive microbiota, intensive care patients, rectal swab

## Abstract

In microbiota research, whole stool sampling is the conventional approach but can be problematic or infeasible for certain patients. This study aims to validate the use of rectal swabbing as an alternative method for microbiota analysis and determine optimal storage conditions suitable for various clinical settings, including intensive care units. We evaluated different sampling techniques and storage temperatures. Our findings indicated that rectal swabs yield microbiota diversity comparable to whole stool samples. Notably, storage conditions significantly impacted microbiota profiles, with increased *E. coli* and *Enterococcus sp*. quantifications observed at room temperature (RT). Consequently, we recommend immediate refrigeration of rectal swabs to reliably assess aerobic and total microbiota, particularly for patients requiring urgent care, such as antibiotic treatment.

**IMPORTANCE:**

We developed a pragmatic approach to study total and aerobic gut microbiota, applicable in numerous clinical units, such as intensive care or emergency units, where whole stool sampling is often impractical. This approach employs ESwab devices, which are already commonly used in hospitals.

## INTRODUCTION

The human microbiota, a diverse community of microorganisms residing on and in our bodies, is a key area in health research. Particularly, the gut microbiota, mainly influenced by diet and antibiotics, plays a vital role in human health (1, 2). Dysbiosis, characterized by imbalances in microbial composition, has been linked to various diseases, including obesity, inflammatory bowel diseases, and cirrhosis (3–6). Quantitative assessment of aerobic bacteria is also crucial for understanding severe infections. Indeed, dysbiosis in the gut microbiota are linked to infections, such as urinary tract infections caused by extended spectrum betaclactamase (ESBL)-producer *Escherichia coli*, which correlate with elevated ESBL-producer *E. coli* quantities in the digestive tract pre-infection (7). Understanding the interactions between the microbiota and pathogenic agents is of primary importance in the prevention and treatment of multiple diseases, paving the way for novel therapeutic strategies targeting microbiota modulation.

Investigating the gut microbiota in numerous patients presents challenges, especially when it comes to obtaining total fecal samples before therapies that could change microbiota composition. This difficulty is particularly pronounced in severely ill patients who require urgent antibiotic therapy. Indeed, patients arriving in emergency departments or intensive care units do not have time to pass stools before receiving antibiotics. Alterations of the microbiota occur very soon after antibiotic administration (8). Moreover, the bacterial density of rectal swab is associated with clinical exposures (9). Thus, the use of this rectal swab for microbiota studies is an interesting alternative. With this technique, it would be possible to set up a larger number of studies with these patients, especially since they are very often rectally swabbed upon admission as part of their care. Comparisons between rectal swabs and whole stool sampling have shown promise as an alternative, yielding comparable microbial compositions using 16S rRNA analysis (10–12). However, these studies utilized dry swabs, which are now less used. Most laboratories now use swabs with transport media (especially Copan ESwab, like in our group of hospitals, the Assistance publique des hôpitaux de Paris [APHP] in France), compatible with automated processing systems, which also enable faster turnaround times for manual processing. The ESwab and Fecal Swab devices consist of a plastic shaft and a flocked tip, the latter coated with nylon fibers, which facilitate rapid and efficient specimen elution. The media inside these devices are modified Amies media, designed to preserve a wide range of potential microbiological pathogens during transport and storage (copangroup.com). Therefore, dry swabs are increasingly less available. On the contrary, Copan ESwab swabs are widely used in French hospitals, thanks to various publications demonstrating the benefits of these transport media in the protection of bacteria (13–15). Previous evaluations of gut microbiota using FecalSwab highlighted some differences compared with analyses performed on whole stools stored without a conservation medium. However, no evaluations have been conducted using ESwab devices which are frequently used in hospitals (16).

Concerning culturomic-based analysis, other researchers have also investigated the use of rectal swabs, particularly ESwab devices, which maintains the viability of several aero-anaerobic bacteria, such as *Streptococcus pneumoniae* and *Haemophilus influenzae*, as well as strictly anaerobic bacteria, up to 48 h at RT, with even better preservation at refrigerated temperatures (13). Additionally, comparative culturomic studies between ESwab and FecalSwab media have been conducted to assess the viability of various diarrheagenic bacteria under different temperature conditions of storage. These studies found that FecalSwab devices provided better viability for some diarrheagenic strains and reference strains, such as *E. coli* ATCC 25922 and *Enterococcus faecalis* ATCC 29212, under refrigerated and frozen conditions (14) and that overall ESwab and FecalSwab devices are compelling media to recover a lot of aerobic and anaerobic bacteria, especially when brought rapidly to the laboratory (within 24 h) as previously mentioned (13–15). However, these studies did not focus on the viability and quantification of various natural isolates of commensal *Enterobacteriaceae* or *Enterococcus species*, which are some of the most frequent bacteria involved in infections originating from the digestive tract in fragile patients, such as cirrhotic patients (17). Beyond bacterial culture with reference strains, other studies have demonstrated that the ESwab medium can reliably be used to study the vaginal or skin microbiota (18, 19). Thus, the assessment of rectal swabs using ESwab devices (commonly used in hospitals) as a reliable tool for microbiota studies (16SrRNA and culturomic analysis) is crucial, especially for patients in emergency departments or intensive care units.

In this study, we sought to closely simulate the real-world conditions of clinical departments where patients receive care. In practice, several difficulties are encountered: samples are not always refrigerated before reaching the laboratory; collecting complete stool samples prior to treatment can be challenging; and some laboratories lack the resources to perform extra research analyses to routine workflows. In order to improve the future feasibility of studies on microbiota diversity in intensive care and emergency situations, we sought to first validate the sampling method (whole stool *versus* rectal swabs) and storage conditions (RT versus 4°C).

## MATERIALS AND METHODS

### Healthy volunteer sampling

**Fig 1:**
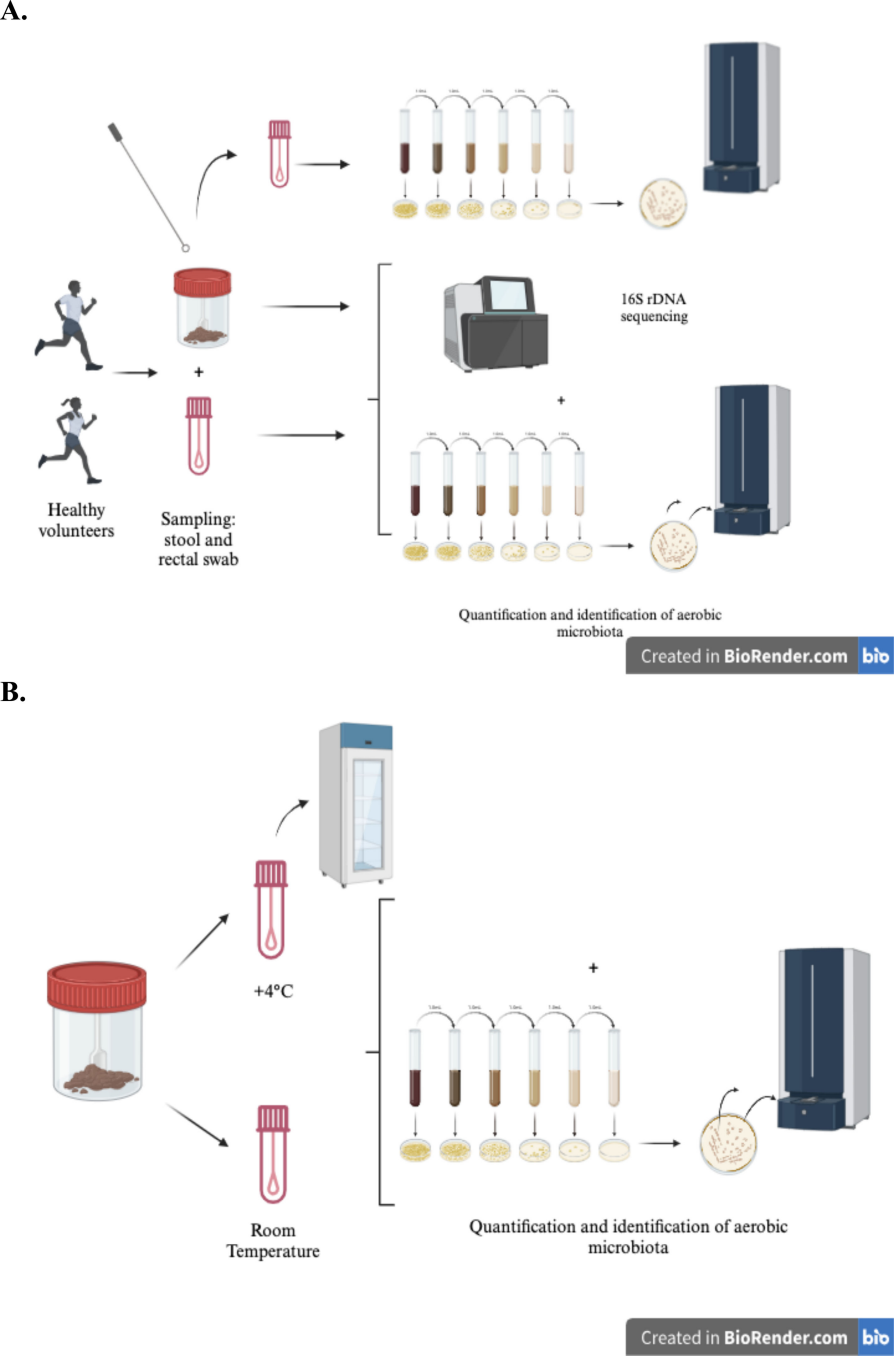
Schematic description of the protocols tested to validate (**A**) the conditions of sampling (total stool/rectal swab) in ESwab medium and (**B**) the temperature condition of storage for the total and aerobic microbiota studies in intensive conditions of clinical practice. Made with BioRender (biorender.com).

Two groups of healthy volunteers were sampled thanks to ethical authorization CLEA number 2019-72. The first group comprised 13 healthy donors who provided fecal samples and rectal swabs. Fecal samples and rectal swabs, self-collected by the volunteers, were directly placed into sterile containers or ESwab devices. Rectal swabs were taken after total stool output within 30 min. Participants brought back their samples (rectal swab and total stool) to the laboratory after unrefrigerated transport within 24 h of stool emission. Once the samples arrived at the laboratory, they were immediately processed for the aerobic microbiota study or frozen in aliquots for the subsequent total microbiota study (Fig. 1A).

Ten other volunteers were sampled and brought their feces after emission to the laboratory within 24 h for further evaluation of temperature storage.

None of the volunteers had taken medication (including antibiotics) or experienced any medical issues in the preceding 6 months.

In accordance with APHP ethics regulations and the Declaration of Helsinki, all participants received comprehensive information and provided consent.

### Evaluation of the rectal swab method for total microbiota sequencing and analysis

#### DNA extraction

We collected 200 mg of total feces or the pellet of liquid contained in the ESwab devices (Copan Diagnostics, Italy) after centrifugation during 10 mn at 5,590×*g*. We then used the DNeasy PowerSoil Pro 250kit (Qiagen, Les Ulis, France) for total manual DNA extraction, following the manufacturer’s protocol.

#### 16SrRNA gene sequencing

Intestinal gut microbiota composition in collected samples was analyzed using 16SrRNA gene sequencing (20) targeting the V4 variable region (21). The manufacturer’s instructions for the library preparation were followed, using KAPAHiFi HotStart ReadyMix (RocheLaboratories, Basel, Switzerland). Briefly, the amplification of the V4 region of the 16S rRNA gene (“PCR1”) was performed with the 515 forward primer (5′-GTGCCAGCMGCCGCGGTAA-3′) and the 806 reverse primer (5′-GGACTACHVGGGTWTCTAAT-3′) as described elsewhere (22). The multiplexing step of the samples on the V4 amplified regions (“PCR2”) required the Nextera Index Kit and NexteraXT Index Kit V2 SetD. PCR products were quantified using the Qubit HS normalized to 4 nM for pooling and sequencing. 16S rRNA gene sequencing was performed with the Illumina MiSeq technology (paired-end reads 2*250 bp). We controlled the quality of the reads with FastQC (23).

#### Pipeline settings

We used the program mothur (24) to process the reads. More specifically, we (i) removed sequences with ambiguous bases, sequences without overlapped regions and sequences longer than 310 bp, (ii) aligned our sequences with the V4 region of the 16S rRNA gene using our primer sequences (SILVA release 128 [25]), (iii) identified and removed chimeric sequences (VSEARCH algorithm [26]), (iv) clustered highly similar sequences with the opticlust algorithm and the cutoff option set to 0.03 (27), (v) determined the taxonomic rank using the SILVA database (release 128 [25]) and computed the relative abundance for each phylotype at the OTU level.

#### Diversity analysis

To be able to compare alpha-diversity estimators among samples and methods, all samples were standardized to the same number of sequences (1,000 sequences) by randomly resampling the operational taxonomic unit (OTU) table (“rarefy_even_depth” function in the R package phyloseq [28]). During this step, five samples were removed because they contained fewer reads than the sample size (1,000 sequences). We conducted alpha-diversity analysis at the OTU level. We computed the Shannon index, which takes into account the number of OTUs and their abundances (“estimate_richness” function in the R package phyloseq [28]). Finally, we evaluated the OTU differential abundance among methods (“DESeq2” function in the R package DESeq [29]).

### Evaluation of the rectal swab method for aerobic microbiota study

#### Comparison of sampling strategies and evaluation of ESwab medium

To compare sampling conditions among 13 healthy volunteers, we estimated bacterial quantification by plating dilutions of suspensions from freshly weighed feces in physiological serum (as the reference) or suspensions from freshly weighed feces in ESwab devices (Copan Diagnostics, Italy), as well as from weighted rectal ESwab devices for each individual, obtained by the difference between the weights of the Eswab devices after and before sampling. These were plated on UriSelect4 plates (Bio-Rad, La Coquette Marches, France), as detailed in Fig. 1A. Aerobic microbiota analysis involved quantifying bacteria in four main classes: *Escherichia coli*, *Enterococcus* species, and other Gram-negative and Gram-positive bacteria species. Main morphologies were confirmed using matrix-assisted laser desorption ionisation time of flight (MALDI-TOF) mass spectrometer (Bruker, Champs-sur-Marne, France) identification before classification into the aforementioned groups. For each condition, WSeS (whole stool in ESwab medium) and RSeS (rectal swab in ESwab), the quantifications of four main groups of bacteria (*E. coli,* other Gram-negative bacteria, *Enterococcus sp*., and *other* Gram-positive bacteria) were in logarithmic scales.

#### Evaluation of storage temperature

To validate the storage temperature conditions post-sampling, we conducted a comparison of *E. coli* and *Enterococcus sp*. quantification after inoculating feces from 10 other healthy volunteers into two ESwab device tubes. One tube was stored at +4°C, while the other was kept at RT. For each tube, we quantified *E. coli* and *Enterococcus sp*. at various time points: initial time (T0), 2 h (T + 2), 4 h (T + 4), 8 h (T + 8), and 24 h (T + 24). Quantification involved plating dilutions of the ESwab medium from each feces on UriSelect4 plates (Bio-Rad, Marne La Coquette, France), as outlined in Fig. 1B. In each case, suspected colonies of *E. coli* and *Enterococcus sp.* were confirmed using MALDI-TOF identification (Bruker, Champs-sur-Marne, France). Then, for each condition of temperature (+4°C and room temperature [RT]) at each time point (T0, T + 2; T + 4, T + 8, and T + 24), we calculated the ratio of logarithms of quantifications of *E. coli* and *Enterococcus sp.* divided by the logarithm of the corresponding quantification at T0, respectively.

### Statistical analyses

All comparisons between groups were performed using a non-parametric test, the Wilcoxon test with Benjamini and Hochberg correction for multiple comparisons when appropriate. To identify OTUs that are differentially detected between samples, we used the Wald test. Quantifications among sampling methods were compared with a pairwise paired Wilcoxon test with Benjamini and Hochberg correction. Association between quantifications (*E. coli* and *Enterococcus sp*.) and time were assessed with Pearson’s correlation tests.

## RESULTS

### Validation of the sampling procedure: evaluation of the rectal swab method for total microbiota study

Overall, for the 13 volunteers, 11 brought back complete sampling (whole stool and rectal swabs), two only brought back whole stool. Regarding 16S rRNA gene analyses, we excluded patient 1 because we did not obtain satisfying sequencing results.

First, we compared sequencing quality data among the three sampling approaches (raw whole stool, whole stool, and rectal swab with ESwab medium conservation) (Table S1). We did not find any differences among sampling approaches, neither for the average sequence length nor for the depth nor for mean sequence quality (pairwise Wilcoxon test, Benjamini and Hochberg correction, *P* > 0.05).

**Fig 2:**
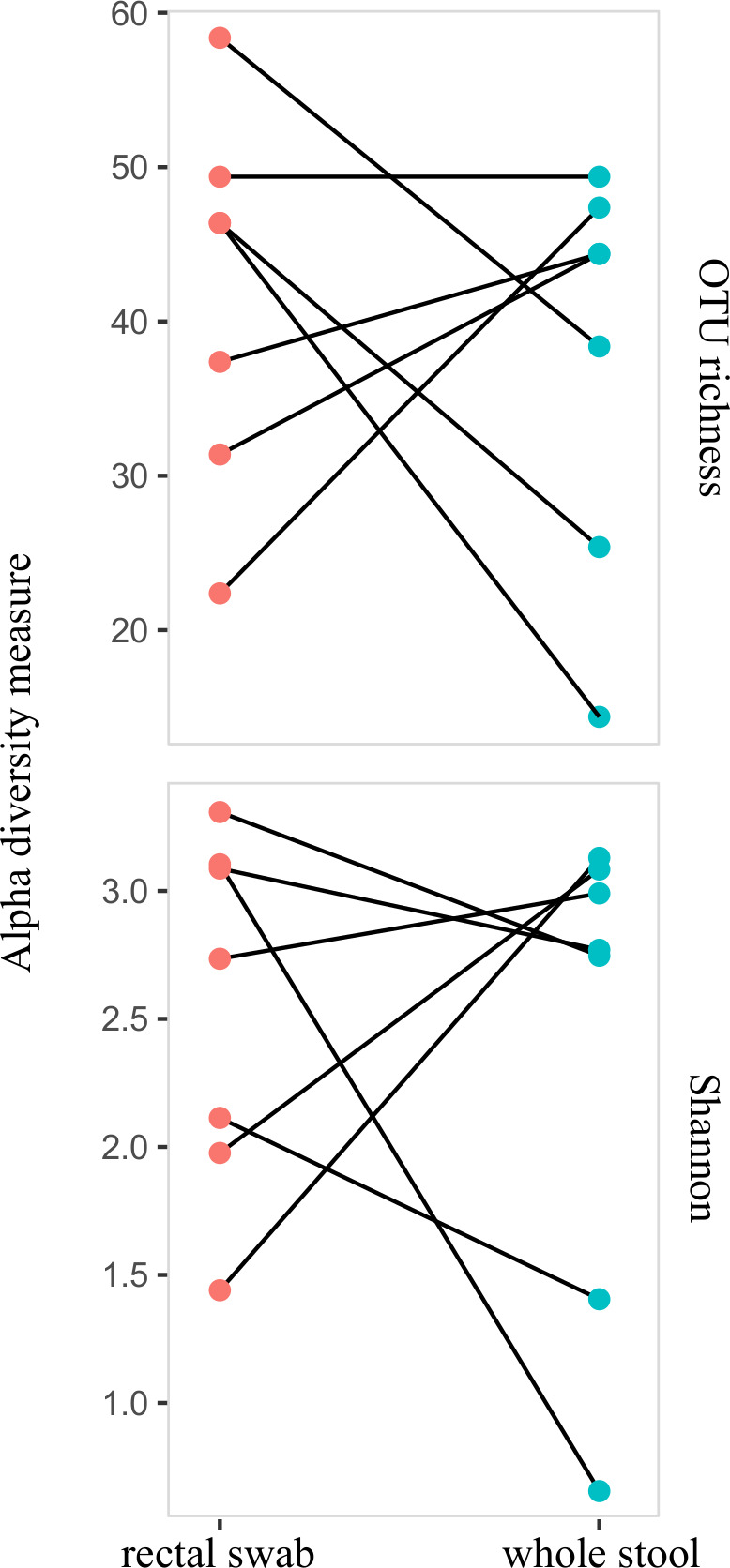
Comparison of the alpha-diversity (OTU richness and Shannon index) among sampling (rectal swab versus whole stool) in ESwab medium.

Then, we compared the OTU richness (alpha-diversity) obtained with two sampling methods, rectal swab, and whole stool, using ESwab medium conservation. We did not find any difference for the alpha-diversity (OTU richness or Shannon index taking into account OTU abundances) between the two sampling methods (paired Wilcoxon test, *P* = 1 for OTU richness and Shannon index, respectively) (Fig. 2). Next, to evaluate the potential changes in community composition between sampling methods, we tested the differences in OTU abundances. With the ESwab medium conservation method, we did not find any difference, with the exception of the genus *Enterococcus*, which was overrepresented (relative abundance of 438.2), in whole stool samples compared with the rectal swab samples (Wald test, *P* = 9.88e-06, base mean = 1689.97, log2 fold change = 12.09) (Table S2).

### Evaluation of the rectal swab method for an aerobic microbiota study

**Fig 3:**
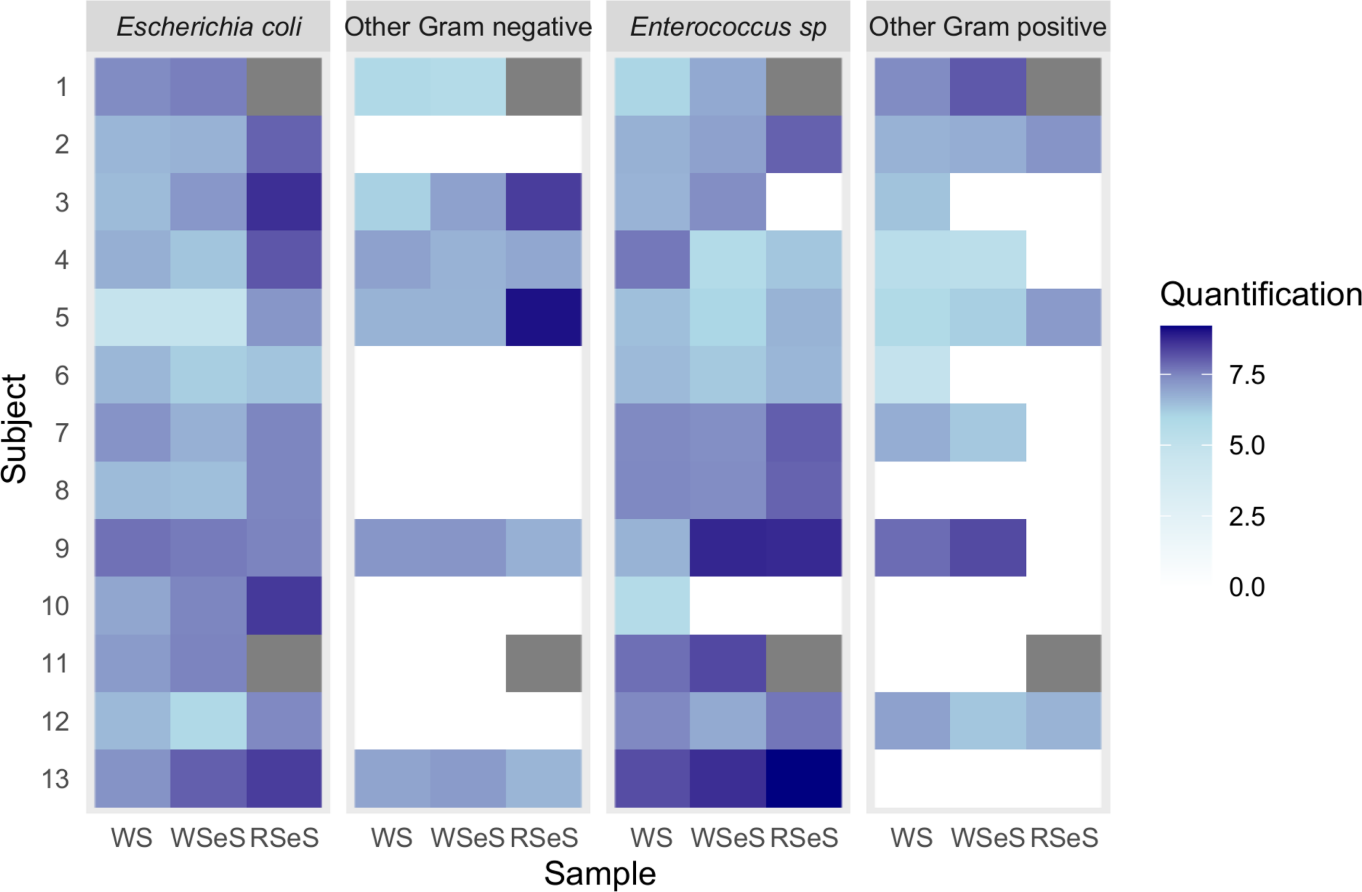
Quantifications of four main groups of bacteria (*Escherichia coli,* other Gram-negative bacteria, *Enterococcus sp*., and other Gram-positive bacteria) for different media and sampling procedures. The quantifications were in logarithmic scales. The different conditions were indicated respectively as WSeS for whole stool in ESwab medium and RSeS for rectal swab in ESwab medium. Missing samples are indicated in gray.

We inoculated whole stool in ESwab medium and compared the quantifications of the four main classes of species between whole stool (considered as the reference), whole stool in ESwab medium (to test the medium effect), and rectal swab in ESwab medium (to evaluate the rectal swabbing effect). Overall, we observed a conservation of all Gram-negative bacteria (*E. coli* and others when present in whole stool) in all conditions. However, we detected a statistically significant increase of *E. coli* quantifications in RSeS (pairwise paired Wilcoxon test with Benjamini–Hochberg correction, *P* = 1.5e-02 and 5.9e-03 when comparing RSeS to WS and to WSeS, respectively). For Gram-positive bacteria, we found at least the species *Enterococcus* in their whole stool. We observed a loss for one and two out of the 11 donors in ESwab medium (for WTeS and RSeS, respectively). We also observed the effect of rectal swabbing with the loss of respectively one *Enterococcus sp*. and other Gram-positive bacteria (*Streptococcus sp.*) for respectively one and three volunteers (Fig. 3; Table S3).

**Fig 4:**
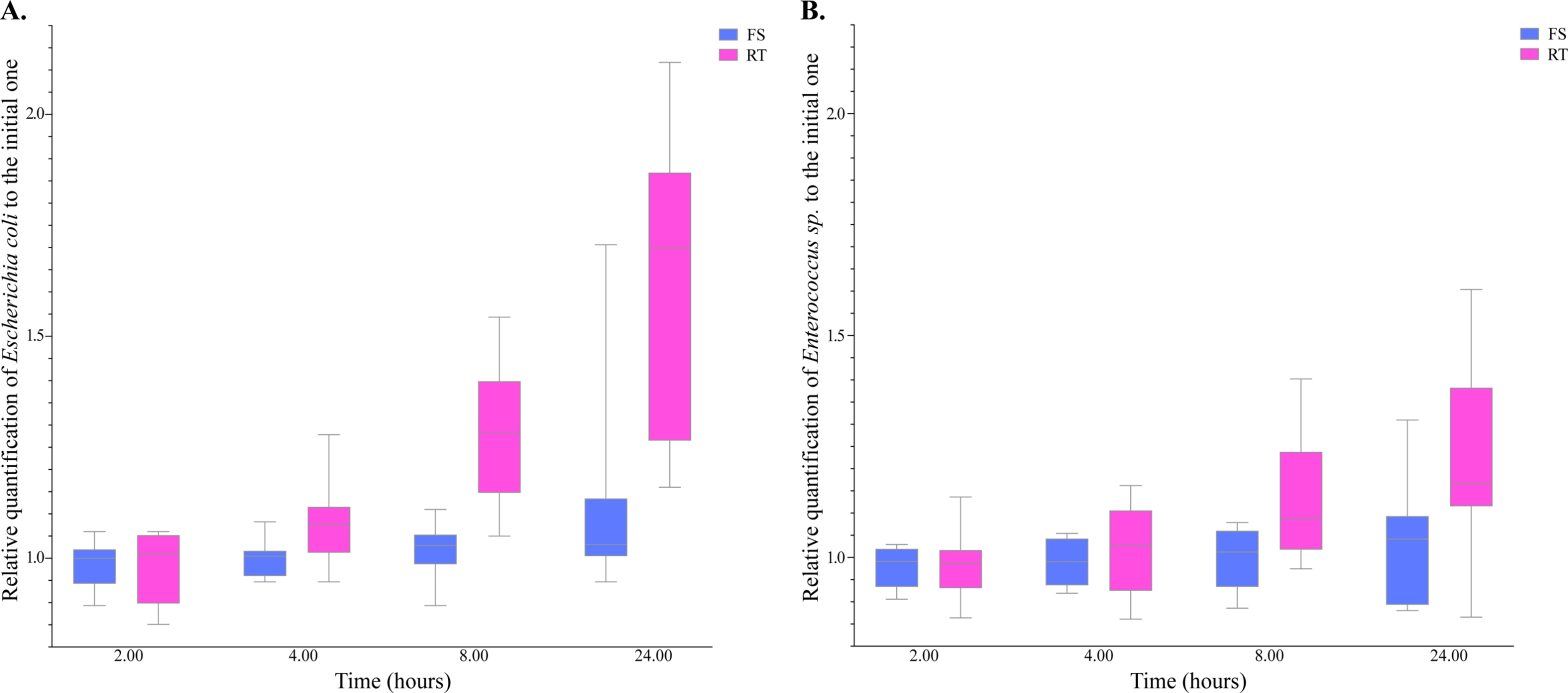
Relative quantifications of *Escherichia coli* and *Enterococcus sp.* across time to the corresponding ones at T0. The quantifications were in logarithmic scales. FS (fridge storage) and room temperature (RT) corresponded to the temperature conditions of storage, +4°C and 20°C.

The increase in *E. coli* quantification with rectal swabs and the fact that healthy volunteers brought their samples (whole stool without any medium and rectal swabs in ESwab medium) without any precaution of storage suggested that these media could have enhanced the multiplication of some bacteria. To test this hypothesis, we performed a comparison of *E. coli* and *Enterococcus sp.* quantifications after inoculating feces from 10 other healthy volunteers into two ESwab devices for two conditions of temperature of storage (+4°C and RT) and quantified those two types of bacteria through time (T0, T + 2, T + 4, T + 8, and T + 24 h) (Fig. 4; Table S4). We observed a significant positive association between *E. coli* quantifications and sampling time at RT (Pearson’s correlation test, r = 0.62, *P* = 1.65e-06). We also detected a positive association, although non-significant, between *Enterococcus sp*. quantifications and sampling time at RT (Pearson’s correlation test, r = 0.25, *P* = 9.24e-02). Quantifications remained stable through time at +4°C for both quantifications of *E. coli* and *Enterococcus sp*. (Pearson’s correlation test, r = 0.04 and 0.07, *P* = 0.78 and 0.63 respectively).

## DISCUSSION

In this study, we aimed to validate the conditions of sampling and conservation methodology for microbiota (total and aerobic) using rectal ESwab devices. Here, we developed a pragmatic approach, applicable in numerous clinical units, such as intensive care or emergency units where obtaining whole stool samples from fragile patients is often impractical.

First, we validated rectal swabbing with ESwab medium for microbiota analysis by comparing sampling conditions and conservation media using samples from 13 healthy volunteers. To our knowledge, this evaluation has never been done before on natural isolates from digestive origin (13, 16). We evaluated bacterial richness (alpha-diversity) obtained from total microbiota analysis and quantifications of aerobic bacteria by aerobic cultures across four main classes: *E. coli*, *Enterococcus sp*., and other Gram-negative and Gram-positive bacteria. We used the Uriselect medium to provide semi-quantitative observation of the aerobic microbiota as a whole, with the aim of identifying an increase in certain potentially pathogenic bacteria. We chose not to use a selective medium in order to minimize the reagents added, and thus have as little impact as possible on routine activity. This inoculation is carried out in the same way as a urine sample and will enable us to identify the majority of bacteria within the aerobic microbiota very easily. We observed a notable increase in *E. coli* quantification with rectal swabbing compared with whole stool sampling. Healthy volunteers performed their swabbing and brought it back to the laboratory. They possibly did not properly conserve the sample at +4°C, which might explain our quantification results.

To investigate this hypothesis, we tested storage conditions and found that samples stored at RT showed significant increases in *E. coli* quantifications, and to a lesser degree in *Enterococcus sp*. quantifications, while quantifications remained stable at 4°C. This finding underscores the importance of maintaining appropriate storage temperatures for accurate bacterial quantifications.

Finally, quantifications and analyses of the differential relative abundance of taxa also indicated a significant decrease in Gram-positive retrieval for bacteria, such as *Enterococcus sp*. and *Streptococcus sp*., under rectal swabbing with ESwab medium. These observations suggest that rectal swabs might be less effective for certain bacterial populations, possibly due to challenges in conservation or sampling efficiency.

Nevertheless, our study demonstrates that rectal swabs can reliably reflect the alpha-diversity (OTU richness and Shannon index) observed in whole stool samples even if we observed some differences for *Enterococcus sp*. in quantifications and in the analyses of the differential relative abundance of taxa. Differences in the relative abundance of bacteria species have also been reported when comparing total stool and fecal transport swabs (FecalSwab, Copan) (16). These differences might be explained by a slight growth disadvantage of certain bacterial taxa in some media, *Enterococcus species* in ESwab medium in our case.

Our study presents several limitations. In addition to the limited number of patients, two of the participants failed to collect samples and did not report rectal swabs. We were not informed of the reason. For future clinical studies, it would be absolutely necessary to remind medical and nurses’ staff to obtain rectal swabs with sufficient fecal materials. Another limitation was the lack of precision regarding the amount of time samples spent at RT before refrigeration However, this absence of refrigeration prior to sample processing by volunteers enabled us to test the limits of what is usually done in hospital patient care conditions.

### Conclusion

The goal of this paper was to closely align with hospital conditions in order to test their limitations and to determine what is feasible or not. Our findings indicate that rectal swabs can be a practical substitute for stool samples in studying both total and aerobic microbiota, and that they could be integrated into routine practice. This decision simplifies procedures in hospital settings where ESwab devices are already commonly utilized. However, careful attention must be given to transport conditions.

## Data Availability

The data generated in this study have been submitted to the European Nucleotide Archive database under accession number PRJEB81899.

## SUPPLEMENTAL MATERIAL

Supplemental tables spectrum.01823-24-s0001.xlsx Tables S1 to S4.

ASM does not own the copyrights to Supplemental Material that may be linked to, or accessed through, an article. The authors have granted ASM a non-exclusive, world-wide license to publish the Supplemental Material files. Please contact the corresponding author directly for reuse.

## References

[1] De Filippo C, Cavalieri D, Di Paola M, Ramazzotti M, Poullet JB, Massart S, Collini S, Pieraccini G, Lionetti P. 2010. Impact of diet in shaping gut microbiota revealed by a comparative study in children from Europe and rural Africa. Proc Natl Acad Sci U S A 107:14691–14696. doi:10.1073/pnas.1005963107 20679230 PMC2930426

[2] Dethlefsen L, Relman DA. 2011. Incomplete recovery and individualized responses of the human distal gut microbiota to repeated antibiotic perturbation. Proc Natl Acad Sci U S A 108 Suppl 1:4554–4561. doi:10.1073/pnas.1000087107 20847294 PMC3063582

[3] Liu J, Wu D, Ahmed A, Li X, Ma Y, Tang L, Mo D, Ma Y, Xin Y. 2012. Comparison of the gut microbe profiles and numbers between patients with liver cirrhosis and healthy individuals. Curr Microbiol 65:7–13. doi:10.1007/s00284-012-0105-8 22484797

[4] Tremaroli V, Bäckhed F. 2012. Functional interactions between the gut microbiota and host metabolism. Nature New Biol 489:242–249. doi:10.1038/nature11552 22972297

[5] Xu M, Wang B, Fu Y, Chen Y, Yang F, Lu H, Chen Y, Xu J, Li L. 2012. Changes of fecal Bifidobacterium species in adult patients with hepatitis B virus-induced chronic liver disease. Microb Ecol 63:304–313. doi:10.1007/s00248-011-9925-5 21814872

[6] Purchiaroni F, Tortora A, Gabrielli M, Bertucci F, Gigante G, Ianiro G, Ojetti V, Scarpellini E, Gasbarrini A. 2013. The role of intestinal microbiota and the immune system. Eur Rev Med Pharmacol Sci 17:323–333. 23426535

[7] Ruppé E, Lixandru B, Cojocaru R, Büke C, Paramythiotou E, Angebault C, Visseaux C, Djuikoue I, Erdem E, Burduniuc O, El Mniai A, Marcel C, Perrier M, Kesteman T, Clermont O, Denamur E, Armand-Lefèvre L, Andremont A. 2013. Relative fecal abundance of extended-spectrum-β-lactamase-producing Escherichia coli strains and their occurrence in urinary tract infections in women. Antimicrob Agents Chemother 57:4512–4517. doi:10.1128/AAC.00238-13 23836184 PMC3754361

[8] Ramirez J, Guarner F, Bustos Fernandez L, Maruy A, Sdepanian VL, Cohen H. 2020. Antibiotics as major disruptors of gut microbiota. Front Cell Infect Microbiol 10:572912. doi:10.3389/fcimb.2020.572912 33330122 PMC7732679

[9] Chanderraj R, Brown CA, Hinkle K, Falkowski N, Woods RJ, Dickson RP. 2022. The bacterial density of clinical rectal swabs is highly variable, correlates with sequencing contamination, and predicts patient risk of extraintestinal infection. Microbiome 10:2. doi:10.1186/s40168-021-01190-y 34991717 PMC8734160

[10] Choudhury R, Middelkoop A, Bolhuis JE, Kleerebezem M. 2019. Legitimate and reliable determination of the age-related intestinal microbiome in young piglets; rectal swabs and fecal samples provide comparable insights. Front Microbiol 10:1886. doi:10.3389/fmicb.2019.01886 31474964 PMC6702655

[11] Fair K, Dunlap DG, Fitch A, Bogdanovich T, Methé B, Morris A, McVerry BJ, Kitsios GD. 2019. Rectal swabs from critically ill patients provide discordant representations of the gut microbiome compared to stool samples. mSphere 4:e00358-19. doi:10.1128/mSphere.00358-19 31341070 PMC6656869

[12] Radhakrishnan ST, Gallagher KI, Mullish BH, Serrano-Contreras JI, Alexander JL, Miguens Blanco J, Danckert NP, Valdivia-Garcia M, Hopkins BJ, Ghai A, Ayub A, Li JV, Marchesi JR, Williams HRT. 2023. Rectal swabs as a viable alternative to faecal sampling for the analysis of gut microbiota functionality and composition. Sci Rep 13:493. doi:10.1038/s41598-022-27131-9 36627399 PMC9831010

[13] Van Horn KG, Audette CD, Sebeck D, Tucker KA. 2008. Comparison of the copan ESwab system with two amies agar swab transport systems for maintenance of microorganism viability. J Clin Microbiol 46:1655–1658. doi:10.1128/JCM.02047-07 18353935 PMC2395074

[14] Hirvonen JJ, Kaukoranta S-S. 2014. Comparison of FecalSwab and ESwab devices for storage and transportation of Diarrheagenic bacteria. J Clin Microbiol 52:2334–2339. doi:10.1128/JCM.00539-14 24740083 PMC4097757

[15] Pichon M, Gebeile R, Lina B, Jacquet G, Gaymard A. 2019. Which sample for the transport of mycoplasma, eSwab or dry swab?, p 95–98. In Annales de biologie clinique10.1684/abc.2018.1407 30799304

[16] Tedjo DI, Jonkers D, Savelkoul PH, Masclee AA, van Best N, Pierik MJ, Penders J. 2015. The effect of sampling and storage on the fecal microbiota composition in healthy and diseased subjects. PLoS One 10:e0126685. doi:10.1371/journal.pone.0126685 26024217 PMC4449036

[17] Ding X, Yu Y, Chen M, Wang C, Kang Y, Lou J. 2019. Causative agents and outcome of spontaneous bacterial peritonitis in cirrhotic patients: community-acquired versus nosocomial infections. BMC Infect Dis 19:463. doi:10.1186/s12879-019-4102-4 31122192 PMC6533661

[18] Bjerre RD, Hugerth LW, Boulund F, Seifert M, Johansen JD, Engstrand L. 2019. Effects of sampling strategy and DNA extraction on human skin microbiome investigations. Sci Rep 9:17287. doi:10.1038/s41598-019-53599-z 31754146 PMC6872721

[19] Mattei V, Murugesan S, Al Hashmi M, Mathew R, James N, Singh P, Kumar M, Lakshmanan AP, Terranegra A, Al Khodor S, Tomei S. 2019. Evaluation of methods for the extraction of microbial DNA from vaginal swabs used for microbiome studies. Front Cell Infect Microbiol 9:197. doi:10.3389/fcimb.2019.00197 31245304 PMC6563847

[20] Yarza P, Yilmaz P, Pruesse E, Glöckner FO, Ludwig W, Schleifer K-H, Whitman WB, Euzéby J, Amann R, Rosselló-Móra R. 2014. Uniting the classification of cultured and uncultured bacteria and archaea using 16S rRNA gene sequences. Nat Rev Microbiol 12:635–645. doi:10.1038/nrmicro3330 25118885

[21] Bukin YuS, Galachyants YuP, Morozov IV, Bukin SV, Zakharenko AS, Zemskaya TI. 2019. The effect of 16S rRNA region choice on bacterial community metabarcoding results. Sci Data 6:1–14. doi:10.1038/sdata.2019.7 30720800 PMC6362892

[22] Caporaso JG, Lauber CL, Walters WA, Berg-Lyons D, Lozupone CA, Turnbaugh PJ, Fierer N, Knight R. 2011. Global patterns of 16S rRNA diversity at a depth of millions of sequences per sample. Proc Natl Acad Sci U S A 108 Suppl 1:4516–4522. doi:10.1073/pnas.1000080107 20534432 PMC3063599

[23] Andrews S. 2017. FastQC: a quality control tool for high throughput sequence data, p 2010

[24] Schloss PD. 2020. Reintroducing mothur: 10 years later. Appl Environ Microbiol 86:e02343-19. doi:10.1128/AEM.02343-19 31704678 PMC6952234

[25] Quast C, Pruesse E, Yilmaz P, Gerken J, Schweer T, Yarza P, Peplies J, Glöckner FO. 2013. The SILVA ribosomal RNA gene database project: improved data processing and web-based tools. Nucleic Acids Res 41:D590–D596. doi:10.1093/nar/gks1219 23193283 PMC3531112

[26] Rognes T, Flouri T, Nichols B, Quince C, Mahé F. 2016. VSEARCH: a versatile open source tool for metagenomics. PeerJ 4:e2584. doi:10.7717/peerj.2584 27781170 PMC5075697

[27] Westcott SL, Schloss PD. 2017. OptiClust, an improved method for assigning amplicon-based sequence data to operational taxonomic units. mSphere 2:e00073-17. doi:10.1128/mSphereDirect.00073-17 28289728 PMC5343174

[28] McMURDIE PJ, Holmes S. 2011. Phyloseq: a bioconductor package for handling and analysis of high-throughput phylogenetic sequence data, p 235–246. In Biocomputing 2012. WORLD SCIENTIFIC, Kohala Coast, Hawaii, USA. PMC3357092 22174279

[29] Love MI, Huber W, Anders S. 2014. Moderated estimation of fold change and dispersion for RNA-seq data with DESeq2. Genome Biol 15:550. doi:10.1186/s13059-014-0550-8 25516281 PMC4302049

